# Application of Matrix-Assisted Laser Desorption/Ionization Time-of-Flight Mass Spectrometry for the Rapid Identification of Yeast Species From Polar Regions

**DOI:** 10.3389/fmicb.2022.832893

**Published:** 2022-02-23

**Authors:** Chenyang He, Jianju Feng, Jing Su, Tao Zhang, Liyan Yu

**Affiliations:** China Pharmaceutical Culture Collection, Institute of Medicinal Biotechnology, Chinese Academy of Medical Sciences and Peking Union Medical College, Beijing, China

**Keywords:** yeasts, Arctic, Antarctic, MALDI-TOF MS, identification

## Abstract

Protein profiling based on matrix-assisted laser desorption/ionization time-of-flight (MALDI-TOF) mass spectrometry (MS) has proved to be a powerful tool for yeast identification. However, it is rarely used in the identification of yeast isolates from polar regions, which may be due to the limited data available for the differentiation of polar yeast species. The present study constructed a supplementary database of MALDI-TOF MS, including 33 yeast species from the Arctic and Antarctica. These yeast species were used to assess the accuracy and practicality of MALDI-TOF MS-based identification compared to the ribosomal DNA [internal transcribed spacer (ITS) and large subunit (LSU) gene regions] sequencing identification. Their dendrogram based on main spectra profiles (MSPs) in the supplementary database was somewhat consistent with their phylogenetic tree. The accuracy of MALDI-TOF MS identification was also compared by the ethanol-formic acid extraction method and the on-plate extraction method. In addition, peptide markers of some yeast species (e.g., *Glaciozyma*, *Phenoliferia*, *Mrakia*, and *Vishniacozyma*) were identified. It is concluded that the MALDI-TOF MS method can differentiate some closely related yeast species from polar regions, thus is suitable for the identification of polar yeasts.

## Introduction

The Arctic and Antarctica have always been the hotspot areas to study the diversity of cold-adapted microorganisms, including yeasts (i.e., a versatile group of eukaryotic organisms; Buzzini et al., 2017). Polar yeasts can withstand the stress conditions in polar regions (e.g., low temperature, desiccation, low levels of nutrients, and UV irradiation), as they have evolved a set of structural and functional adaptation strategies to thrive in these extreme environments, such as production of cold-active enzymes, anti-freeze compounds, and extracellular polymers (Buzzini et al., 2012; Nizovoy et al., 2021). In recent years, it was found that polar yeasts with unique phenotypic characteristics had great biotechnological potentials, such as the production of cold-active enzymes (e.g., cellulase, amylase, protease, and lipase; Barahona et al., 2016; Tsuji, 2017; Wang et al., 2019) and bioactive compounds (e.g., exopolysaccharide; Pavlova et al., 2009; Viñarta et al., 2020), degradation of pollutant (e.g., phenol-related compounds and petroleum hydrocarbons) at low temperature (Fernandez et al., 2017; Martínez-Ávila et al., 2021), and potential probiotic (Coutinho et al., 2021a,b). Besides, some polar yeasts (e.g., *Aureobasidium pullulans*) may cause opportunistic infections and become potential pathogens to humans (Buzzini et al., 2017).

The traditional yeast identifications based on morphological, physiological, and biochemical characteristics are time-consuming and the conclusion may be unreliable due to lack of experience or subjective judgment (Agustini et al., 2014). The molecular method using the ribosomal DNA [internal transcribed spacer (ITS) and large subunit (LSU) gene regions] sequencing has been widely applied to polar yeast identification (Pathan et al., 2010; Zhang et al., 2014). In recent studies, multigene phylogenetic analyses based on DNA sequencing were used to identify novel yeast species isolated from various environments (Liu et al., 2015; Wang et al., 2015; Li et al., 2020). Although the molecular method is regarded as the gold standard in yeast identification, it is relatively time-consuming as compared to matrix-assisted laser desorption/ionization time-of-flight (MALDI-TOF) mass spectrometry (MS) analysis (Moothoo-Padayachie et al., 2013; Paul et al., 2019). Therefore, rapid and accurate identification of yeasts is preferred, especially in clinical, agricultural, and industrial applications (Spitaels et al., 2015; Jin et al., 2020; Martins et al., 2020). The accuracy of the MALDI-TOF MS based microbial identification has been proven to be comparable to automated systems based on biochemical and other phenotypic tests (Florio et al., 2018). In addition, MALDI-TOF MS was effective for identifying yeast isolated from food (e.g., *Candida* and *Debaryomyces* species; Pavlovic et al., 2014), beverages (e.g., *Saccharomyces* species; Usbeck et al., 2014; Zhang et al., 2020), and clinical samples (e.g., *Candida* and *Cryptococcus* species; Tan et al., 2012; Patel, 2019; Pote et al., 2020).

Identification of polar yeast species is a challenge as the current MALDI-TOF MS database of yeast species is limited that spectra profiles of polar yeast species are not included, leading to their misidentification as related species. To the best of our knowledge, there was only one study on the identification of psychrophilic yeast using MALDI-TOF MS, i.e., Dalluge et al. (2019) employed MALDI-TOF MS to rapidly characterize and distinguish three psychrophilic yeasts (*Rhodotorula mucilaginosa*, *Naganishia vishniacii*, and *Dioszegia cryoxerica*) from three mesophilic yeasts (*Saccharomyces cerevisiae* strain WLP862, *S. cerevisiae* strain WLP051, and *S. pastorianus*). The present study aimed to (1) construct a supplementary database with spectra of the 33 polar yeast species, (2) compare molecular analysis and MALDI-TOF MS analysis for yeast species differentiation, (3) compare ethanol-formic acid extraction method and on-plate extraction method for MALDI-TOF MS identification, and (4) determine the peptide markers of polar yeasts.

## Materials and Methods

### Yeast Strains

A total of 33 yeast species were isolated from various environments in the Arctic and Antarctica (Table 1). Among them, 22 yeast species were isolated from the Ny-Alesund Region (78°55′N, 11°56′E) located in the Svalbard Islands (high Arctic), and 11 species were isolated from the King George Island (62°23′S, 58°27′W) located in the South Shetland Islands (maritime Antarctica). All the yeast strains were deposited in the China Pharmaceutical Culture Collection (CPCC),^1^ Institute of Medicinal Biotechnology, Chinese Academy of Medical Sciences & Peking Union Medical College.

**Table 1:** Information on the origins of the 33 polar yeast species and results of molecular identification and matrix-assisted laser desorption/ionization time-of-flight mass spectrometry (MALDI-TOF MS)-based identification.

Test strains	Geographical origins	Environmental origins	Molecular biology analysis closest type strain		MALDI-TOF MS-based identification (score A^*^/score B^#^)
			ID by LSU (similarity %)	ID by ITS (similarity %)	
**Saccharomycetes**					
CPCC 300416	Arctic	Sea water	*Candida davisiana* (99.67%)	*Candida davisiana* (99.26%)	*Candida davisiana* (9.720/9.228)
CPCC 300347	Antarctic	Dung	*Candida glaebosa* (99.32%)	*Candida glaebosa* (99.19%)	*Candida glaebosa* (9.742/9.510)
CPCC 300486	Antarctic	Sea water	*Galactomyces geotrichum* (99.83%)	*Galactomyces geotrichum* (99.68%)	*Galactomyces geotrichum* (9.836/9.002)
CPCC 300461	Antarctic	Sea water	*Metschnikowia australis* (99.59%)	*Metschnikowia australis* (99.15%)	*Metschnikowia australis* (9.585/9.028)
CPCC 300438	Arctic	Sea water	*Metschnikowia bicuspidata* (99.61%)	*Metschnikowia bicuspidata* (97.74%)	*Metschnikowia bicuspidata* (9.268/9.035)
CPCC 300437	Arctic	Sea water	*Metschnikowia zobellii* (100%)	*Metschnikowia zobellii* (98.38%)	*Metschnikowia zobellii* (9.539/9.303)
**Cystobasidiomycetes**					
CPCC 300370	Arctic	Dung	*Cystobasidium laryngis* (100%)	*Cystobasidium laryngis* (99.45%)	*Cystobasidium laryngis* (9.612/9.426)
CPCC 300329	Antarctic	Soil	*Cystobasidium ongulense* (99.84%)	*Cystobasidium ongulense* (99.30%)	*Cystobasidium ongulense* (9.731/9.096)
**Microbotryomycetes**					
CPCC 300487	Antarctic	Soil	*Glaciozyma antarctica* (100%)	*Glaciozyma antarctica* (99.89%)	*Glaciozyma antarctica* (9.788/9.515)
CPCC 300440	Arctic	Sea water	*Glaciozyma litoralis* (100%)	*Glaciozyma litoralis* (99.75%)	*Glaciozyma litoralis* (9.707/9.562)
CPCC 300358	Antarctic	Soil	*Glaciozyma martinii* (100%)	*Glaciozyma martinii* (100%)	*Glaciozyma martinii* (9.692/9.559)
CPCC 300392	Arctic	Soil	*Glaciozyma watsonii* (100%)	*Glaciozyma watsonii* (99.67%)	*Glaciozyma watsonii* (9.802/9.447)
CPCC 300366	Arctic	Plant	*Leucosporidium muscorum* (100%)	*Leucosporidium muscorum* (98.93%)	*Leucosporidium muscorum* (9.209/6.283)
CPCC 300410	Arctic	Plant	*Leucosporidium scottii* (100%)	*Leucosporidium scottii* (99.84%)	*Leucosporidium scottii* (9.484/9.283)
CPCC 300463	Antarctic	Soil	*Phenoliferia glacialis* (100%)	*Phenoliferia glacialis* (99.34%)	*Phenoliferia glacialis* (9.676/9.107)
CPCC 300464	Antarctic	Soil	*Phenoliferia psychrophenolica* (99.83%)	*Phenoliferia psychrophenolica* (99.32%)	*Phenoliferia psychrophenolica* (9.710/9.552)
CPCC 300465	Antarctic	Soil	*Phenoliferia psychrophila* (99.68%)	*Phenoliferia psychrophila* (99.67%)	*Phenoliferia psychrophila* (9.241/6.232)
**Tremellomycetes**					
CPCC 300411	Arctic	Dung	*Cystofilobasidium macerans* (99.69%)	*Cystofilobasidium macerans* (99.68%)	*Cystofilobasidium macerans* (9.777/9.555)
CPCC 300426	Antarctic	Lichen	*Dioszegia hungarica* (99.36%)	*Dioszegia hungarica* (99.19%)	*Dioszegia hungarica* (9.609/9.548)
CPCC 300381	Arctic	Feather	*Filobasidium magnum* (99.53%)	*Filobasidium magnum* (99.82%)	*Filobasidium magnum* (9.703/8.483)
CPCC 300369	Arctic	Dung	*Goffeauzyma gastrica* (100%)	*Goffeauzyma gastrica* (99.68%)	*Goffeauzyma gastrica* (9.621/9.515)
CPCC 300368	Arctic	Fresh water	*Holtermanniella nyarrowii* (99.52%)	*Holtermanniella nyarrowii* (99.48%)	*Holtermanniella nyarrowii* (9.624/9.534)
CPCC 300345	Arctic	Plant	*Mrakia aquatica* (100%)	*Mrakia aquatica* (98.75%)	*Mrakia aquatica* (9.722/9.585)
CPCC 300333	Antarctic	Lichen	*Mrakia blollopis* (99.68%)	*Mrakia blollopis* (98.89%)	*Mrakia blollopis* (9.701/9.590)
CPCC 300344	Arctic	Plant	*Mrakia niccombsii* (100%)	*Mrakia niccombsii* (100%)	*Mrakia niccombsii* (9.528/9.531)
CPCC 300477	Arctic	Soil	*Mrakia robertii* (99.84%)	*Mrakia robertii* (99.36%)	*Mrakia robertii* (9.590/9.412)
CPCC 300377	Arctic	Feather	*Naganishia albidosimilis* (99.83%)	*Naganishia albidosimilis* (100%)	*Naganishia albidosimilis* (9.734/5.059)
CPCC 300436	Arctic	Sea water	*Naganishia diffluens* (99.84%)	*Naganishia diffluens* (100%)	*Naganishia diffluens* (9.681/9.063)
CPCC 300475	Arctic	Sea water	*Naganishia friedmannii* (99.68%)	*Naganishia friedmannii* (99.34%)	*Naganishia friedmannii* (9.510/9.120)
CPCC 300478	Arctic	Soil	*Piskurozyma fildesensis* (99.84%)	*Piskurozyma fildesensis* (99.84%)	*Piskurozyma fildesensis* (9.563/9.291)
CPCC 300372	Arctic	Fresh water	*Vishniacozyma carnescens* (99.20%)	*Vishniacozyma carnescens* (99.63%)	*Vishniacozyma carnescens* (9.606/9.320)
CPCC 300352	Arctic	Plant	*Vishniacozyma tephrensis* (99.67%)	*Vishniacozyma tephrensis* (100%)	*Vishniacozyma tephrensis* (9.600/9.208)
CPCC 300476	Arctic	Feather	*Vishniacozyma victoriae* (100%)	*Vishniacozyma victoriae* (100%)	*Vishniacozyma victoriae* (9.720/9.453)

* Score A: identification score using ethanol-formic acid extraction method.

# Score B: identification score using on-plate extraction method.

### DNA Extraction, Amplification, and Sequencing

Genomic DNA of yeasts was extracted with a modified Chelex-100 boiling method (Walsh et al., 1991). The yeast colony was placed into a 1.5 ml centrifuge tube containing 100 μl Chelex 100 resin solution [10% (wt/vol) in water; Bio-Rad Laboratories, United States]. The mixture was vortexed and heated in a microwave oven for 4 min. After centrifuging at 13,000 rpm for 4 min, the supernatant was used for the subsequent PCR amplification. The D1/D2 domains of the LSU ribosomal DNA and the ITS region of the ribosomal DNA were amplified with the target primers NL1 (5′-GCATATCAATAAGCGGAGGAAAAG-3′) and NL4 (5′-GGTCCGTGTTTCAAGACGG-3′; Kurtzman and Robnett, 1991), ITS1F (5′-CTTGGTCATTTAGAGGAAGTAA-3′; Gardes and Bruns, 1993) and ITS4 (5′-TCCTCCGCTTATTGATATGC-3′; White et al., 1990), respectively. The PCR mixture (50 μl): Easy Taq Buffer 6.0 μl, Easy Taq DNA Polymerase 1.5 μl, dNTP 4.0 μl, target primer pairs 1.5 μl for each one, ddH_2_O 33.5 μl. Amplification of the D1/D2 domain was performed as follows: 94°C for 6 min, followed by 40 cycles of 94°C for 1 min, 50°C for 1 min, and 72°C for 1 min, and concluded with a final elongation step at 72°C for 5 min. The PCR amplification of ITS region was as follows: 95°C for 3 min, followed by 37 cycles of 94°C for 30 s, 52°C for 30 s, and 72°C for 40 s, and a final elongation step at 72°C for 10 min. The sequence data of D1/D2 domains were deposited in the GenBank database under the accession numbers MZ686226-MZ686249 and MZ686251-MZ686259, whereas sequence data of ITS region under the accession numbers MZ683224-MZ683247 and MZ683249-MZ683257.

### Molecular Identification and Phylogenetic Analysis

The yeast species were identified based on sequence similarity (i.e., the percentage of nucleotide identity with reference sequences) and the phylogenetic position. For sequence similarity determination, blastn searches were performed in the GenBank public sequence databases to find the closest related species.

For phylogenetic analysis, the sequences of D1/D2 domain and ITS region were aligned using MAFFT^2^ respectively. Some ambiguous sites were manually inspected in BioEdit version 7.0.9.0. ModelFinder of PhyloSuite version 1.1.16 was used to select the best-fit model (GTR + G + I) using Akaike information criteria (AIC) for DNA substitution (Kalyaanamoorthy et al., 2017). A total of 1,072 characters (including gaps) were obtained. Maximum likelihood (ML) analysis was conducted by RAxML-HPC version 8.2.10 with 1,000 bootstrap replicates (Stamatakis, 2014). The phylogenetic tree was visualized and edited in FigTree version 1.4.4 (Rambaut, 2018), and bootstrap values ≥ 50% are shown above branches.

### Construction of MALDI-TOF MS Supplementary Database

All the 33 yeast species were cultured on yeast malt agar (YMA; Difco, Becton-Dickinson, United States) at 12°C for 4–5 days. For supplementary database implementation, the main spectra profiles (MSPs) of each yeast species were created using three replicates of eight separated colonies from each yeast species to be included in the user-generated library.

Each yeast colony was extracted according to the ethanol-formic acid extraction method. The yeast colony was placed into a 1.5 ml centrifuge tube and mixed with 300 μl of ddH_2_O and 900 μl of absolute ethanol. The tubes were centrifuged at room temperature (14,000 rpm, 4 min). After the supernatant was discarded, the obtained pellet was air-dried. Subsequently, 10 μl of 70% formic acid (Lysis Solution 1, Autobio Diagnostics, China) and 10 μl of acetonitrile (Lysis Solution 2, Autobio Diagnostics, China) were added and mixed entirely in the tubes. After centrifugation at 14,000 rpm for 4 min at room temperature, 1.0 μl of the supernatant was spotted in eight replicates onto a 96-spot polished steel target plate (Autobio Diagnostics, China) and air-dried at room temperature. Then, each spot was overlaid with 1.0 μl of the matrix solution (α-cyano-4-hydroxycinnamic acid; Autobio Diagnostics, China) on each spot and air-dried completely before MALDI-TOF MS measurement.

Prior to MALDI-TOF MS analysis, the mass spectrometer was externally calibrated using calibrating agent (containing *Escherichia coli* DH5α, Ribonuclease, and Myoglobin; Autobio Diagnostics, China) with a protein mix of E.PM 3K^+^ (3637.22 Da), E.PM 4K^+^ (4365.343 Da), E.PM 5K^+^ (5096.776 Da), E.PM 5.3K^+^ (5381.446 Da), E.PM 6K^+^ (6255.444 Da), E.PM 7K^+^ (7274.467 Da), E.PM 10K^+^ (10300.032 Da), E.PM 13K^+^ (13683.173 Da), and E.PM 16K^+^ (16952.232 Da). Spectra with peptide peaks outside the allowed average were not considered. Spectra profiles were loaded into the Autof Acquirer software Version 2.0.59 (Autobio Diagnostics, China). The MALDI-TOF MS spectra profile of each sample was measured by Autof ms1000 (Autobio Diagnostics, China). Spectral data were taken in the *m/z* range of 2,000–20,000 Da. Pictures of sample-matrix crystals were taken by a built-in camera. The spectral data were collected by software Autof Acquirer version 2.0.59 (Autobio Diagnostics, China) and analyzed by software Autof Analyzer version 2.0.14 (Autobio Diagnostics, China).

The main spectra profiles of each yeast species were generated considering the 24 spectra profiles obtained and were saved in the reference library. This supplementary database of 33 polar yeast species can be found on CPCC, Institute of Medicinal Biotechnology, Chinese Academy of Medical Sciences & Peking Union Medical College.

### MALDI-TOF MS Data Analysis

The MALDI-TOF MS-based clustering analysis was performed using software Autof Analyzer version 2.0.14 (Autobio Diagnostics, China). MALDI mass spectra for individual yeast species from the 33 yeasts studied were collected and catalogued for comparison and identification of genus-specific peptide markers.

### Identification With Supplementary Database Using Two Extraction Methods

The supplementary database was subsequently used for species identification using two extraction methods. All the 33 yeast species were cultured again on YMA (Difco, Becton–Dickinson, United States) at 12°C for 4–5 days. Each yeast sample was extracted using both the ethanol-formic acid extraction method and the on-plate extraction method. For the on-plate extraction method, yeast colonies were directly transferred on the target plates as thin films using sterile inoculation loops. Following this, 1.0 μl of Lysis Solution 1 (70% formic acid) was mixed with the yeast sample on the plate by pipetting, and the resultant mixture was air-dried at room temperature. Finally, 1.0 μl of the matrix solution was applied onto the spot and air-dried completely prior to MALDI-TOF MS measurement. After extraction procedures, all the yeast samples were identified by Autof ms1000, following the manufacturers’ instructions.

For identification, the acquired spectrum was loaded into the Autof Acquirer version 2.0.59 (Autobio Diagnostics, China) and was compared with spectra deposited in the supplementary database as MSPs. The yeast samples were correctly identified to the species level required a score > 9.0; a score between 6.0 and 9.0 indicated genus identification; but a score < 6.0 indicated no significant similarity of the spectrum and unidentified.

## Results and Discussion

### Species Differentiation Using Molecular Analysis and MALDI-TOF MS Analysis

**Figure 1:**
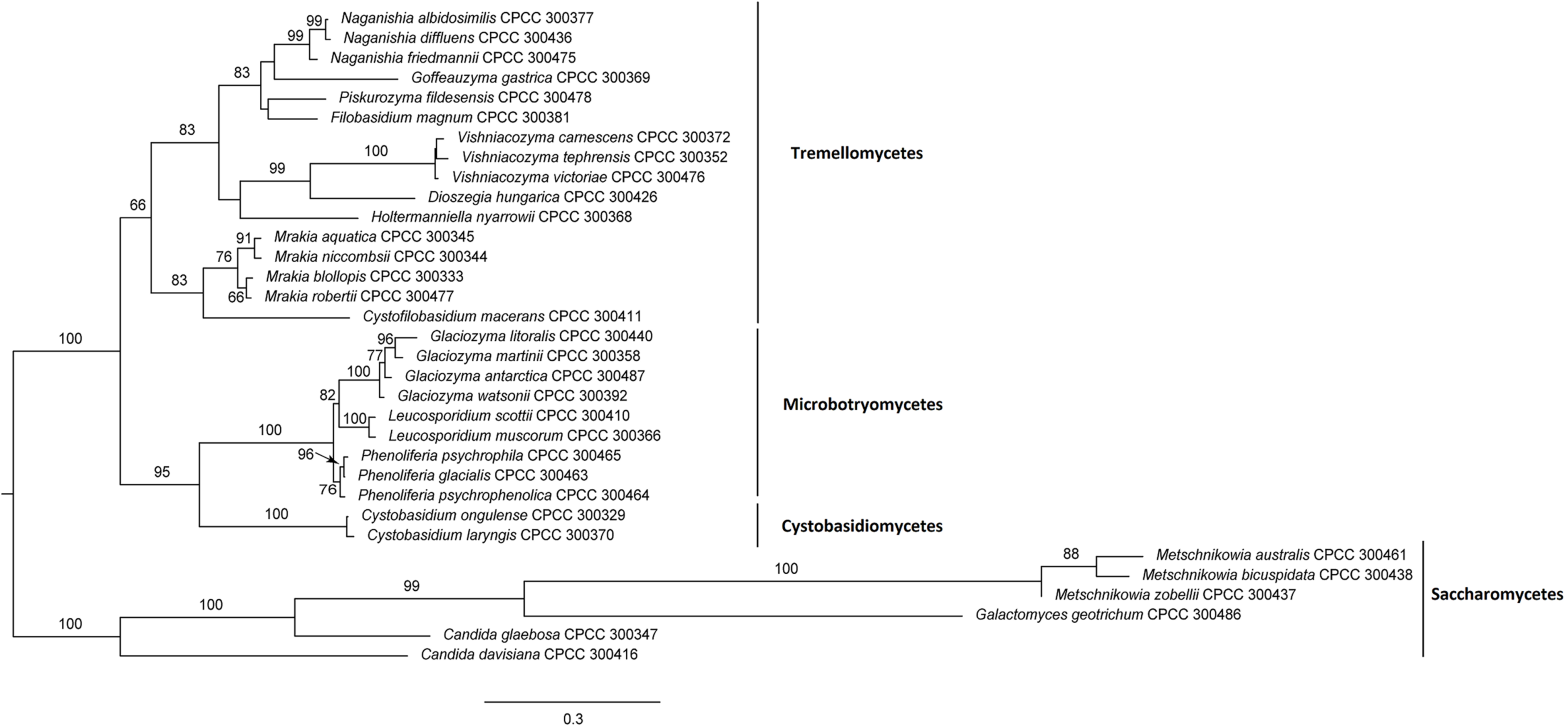
Maximum likelihood (ML) tree inferred from sequences of D1/D2 domain and internal transcribed spacer (ITS) region under the GTR + G + I model in RAxML. ML bootstrap values (≥50%) are indicated along branches (ML).

A total of 33 yeast species were isolated from the Arctic (22 species) and Antarctic (11 species) and were used for sequence similarity analysis (Table 1) and phylogenetic analysis (Figure 1). These 33 yeast species belonged to 16 genera, 13 families, eight orders, four classes, and two phyla. In the present study, a ML phylogenetic tree was constructed using the combined sequences of the LSU rDNA gene and ITS region (Figure 1). The ML phylogenetic tree indicated that 27 yeast species belonged to the phylum Basidiomycota, and six yeast species belonged to the phylum Ascomycota. The Basidiomycota included three classes: Tremellomycetes (16 species), Microbotryomycetes (six species), and Cystobasidiomycetes (two species). The Ascomycota included one class: Saccharomycetes (six species). They were all supported by high bootstrap values (≥50%) in the ML phylogenetic tree.

**Figure 2:**
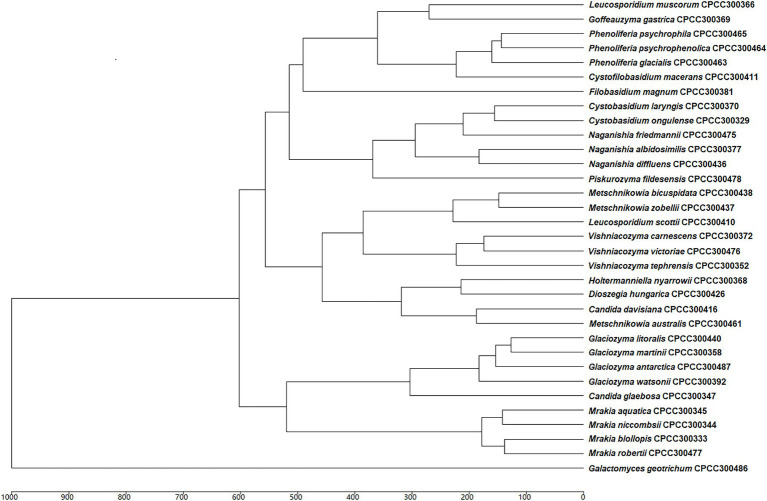
Main spectra profile-based dendrogram of the 33 yeast species included in the supplementary database.

Based on previous molecular identification results, the MALDI-TOF MS supplementary database of the 33 yeast species was constructed based on the 24 spectra profiles for each species (e.g., *Cystobasidium laryngis* CPCC 300370 in Supplementary Figure S1). A hierarchical clustering dendrogram was generated using MSPs of yeast species in the supplementary database constructed (Figure 2). The topology of the clustering dendrogram was then compared with the phylogenetic tree based on sequencing data. The clustering of yeast species in dendrogram was basically consistent with phylogenetic analysis of DNA sequence data. For example, most yeast species (e.g., *Glaciozyma*, *Mrakia*, and *Vishniacozyma*) formed the expected groups in the two trees. However, Tremellomycetes, Microbotryomycetes, Cystobasidiomycetes, and Saccharomycetes clades formed the expected groups within the resulting phylogenetic tree but did not form groups in MALDI-TOF MS-based dendrogram. Wigmann et al. (2019) proposed that MALDI-TOF MS data was inappropriate for phylogenetic analysis, as it was based on the *m/z* values of ribosomal proteins and other proteins but not protein sequences.

### Determination of Peptide Markers of Polar Yeast Species

**Figure 3:**
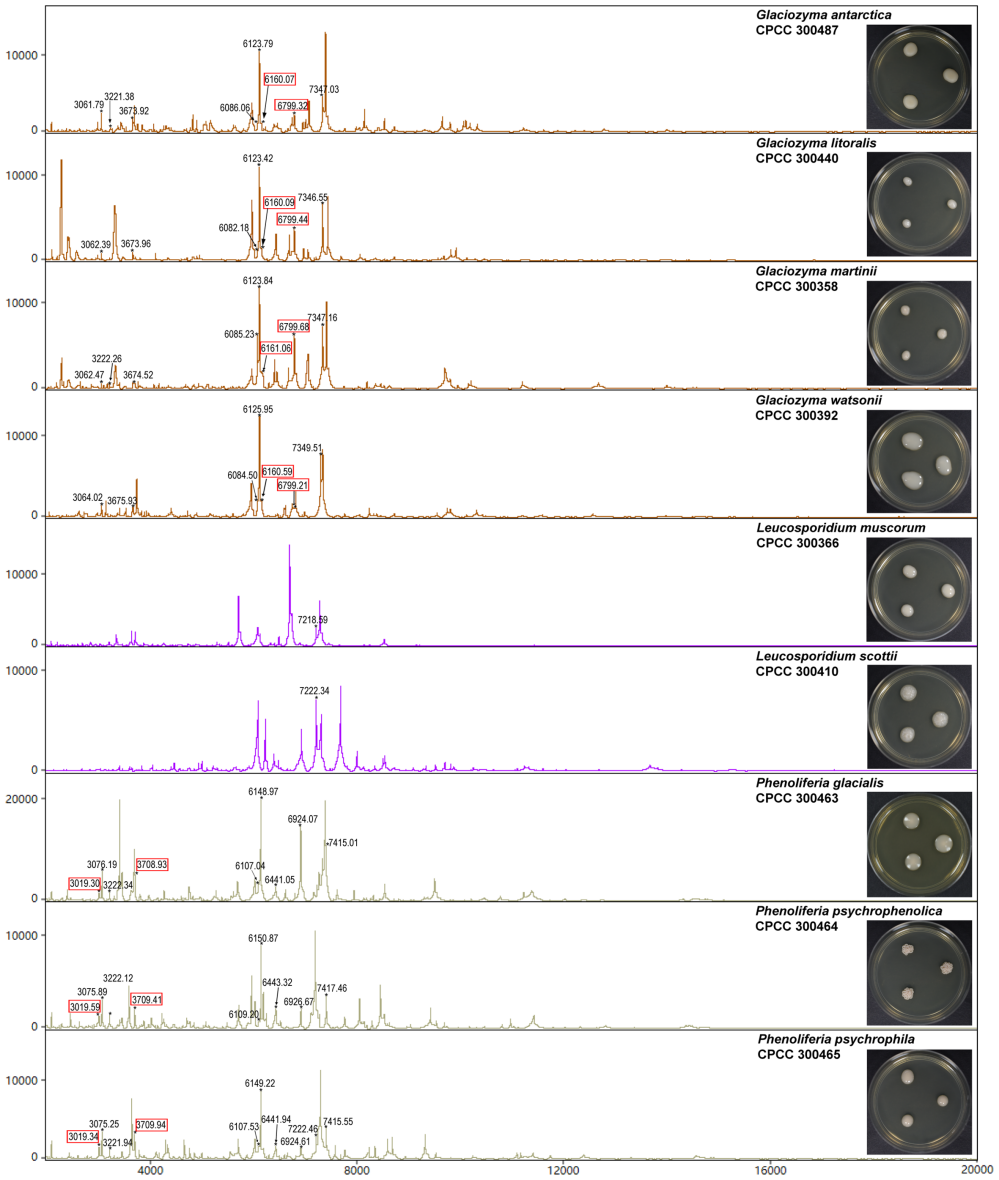
Representative mass spectra of the nine yeast species in Microbotryomycetes. The *Glaciozyma* genus-specific markers (about *m/z* 6160 and 6799 Da) and *Phenoliferia* genus-specific markers (about *m/z* 3019 and 3709 Da) were indicated as the red quadrangles.

The mass spectra of the 33 polar yeasts were included as references in a supplementary MALDI-TOF MS database, and their colonial morphology were also recorded (Figure 3; Supplementary Figures S2, S3). The peptide peaks of all the 33 polar yeast species were compared in the present study, and 15 peptide peaks (*m/z* deviation ≤ 10 Da) were detected in ≥9 yeast species (Table 2). The common peptide peaks of polar yeast species were observed, such as *m/z* 2091.8 and 2104.8 appeared in the four classes and >70% polar yeast species. Furthermore, several peptide peaks (*m/z* deviation ≤ 2 Da) were determined as certain genus-specific markers. For example, *Glaciozyma*-specific markers were *m/z* 6160 and 6799, *Phenoliferia*-specific marker *m/z* 3019 and 3709 (Table 3), *Mrakia*-specific marker *m/z* 5966, *Vishniacozyma*-specific marker *m/z* 5006 (Supplementary Table S1), and *Metschnikowia*-specific marker *m/z* 5256 (Supplementary Table S2). In addition, some peptide peaks in the same genus were not classified as a genus-specific marker (e.g., *m/z* 3061.79–3064.02, *m/z* 3673.92–3675.93, *m/z* 6082.18–6086.06, *m/z* 6123.42–6125.95, and *m/z* 7346.55–7349.51 in *Glaciozyma* spp.), because their *m/z* deviations were above 2 Da (Figure 3).

**Table 2:** Peptide markers of the 33 yeast species from polar regions.

	2091.8	2104.8	3074.4	3224.0	3976.5	4855.8	6029.0	6057.8	6086.2	6151.3	6406.6	6444.0	7346.2	7818.9	8542.3
*Candida davisiana*	+	+	−2.9		−1.6		+	+			+	+	−3.7	+2.0	
*Candida glaebosa*	+	+				+2.2	+3.0		−2.1	+2.2					
*Galactomyces geotrichum*			+	+1.5											
*Metschnikowia australis*	+	+					−2.4	+1.6	+3.0	+1.2	+1.4	−3.6		−4.0	
*Metschnikowia bicuspidata*							+3.1								+2.6
*Metschnikowia zobellii*	+	+	+				+3.1			+				+2.5	
*Cystobasidium laryngis*	+	+	−3.9		−2.1	+3.5	−3.6						−2.0		
*Cystobasidium ongulense*	−1.2	+	+2.5							+2.9		+3.2			
*Glaciozyma antarctica*	+	+		−2.6					−4.1				+		+1.5
*Glaciozyma litoralis*		+4.3							−3.9			+	+		
*Glaciozyma martinii*				−1.7		+			+				+		
*Glaciozyma watsonii*					+				−1.7				+3.3		
*Leucosporidium muscorum*	+	+					+		+4.6						−2.3
*Leucosporidium scottii*						+1.1					−1.1	+1.3		+4.0	
*Phenoliferia glacialis*	+2.0	+1.7	+1.8	−1.7	+2.4					−2.3		−3.0			
*Phenoliferia psychrophenolica*	+	+	+1.5	−1.9	+	−3.6				+		+			+
*Phenoliferia psychrophila*	+	+	+	−2.0						−2.1		−2.0	+		−2.2
*Cystofilobasidium macerans*	+	+		+	+4.6	−2.0	−2.1	+2.8						−3.7	+
*Dioszegia hungarica*	+		−1.9	+4.2	+2.2	−4.2						+3.4		+	
*Filobasidium magnum*	+	−1.2		+3.1		−2.6		+							
*Goffeauzyma gastrica*	+	+			+1.3	+4.1	−2.6	+							
*Holtermanniella nyarrowii*								−1.6	+3.0	+2.6					
*Mrakia aquatica*	−2.4	−2.2				+				−3.6	+			−1.8	+1.2
*Mrakia blollopis*	−1.8	−2.2			+						+				
*Mrakia niccombsii*	−2.1	−1.4		−3.9							+	+		−1.9	+1.1
*Mrakia robertii*	+	+			+					+	+	+	−1.6	+1.7	
*Naganishia albidosimilis*	−1.6	−4.3		+4.5	−4.9										
*Naganishia diffluens*					−3.0			−1.9	−1.9						+
*Naganishia friedmannii*	+	+2.5	+1.4		+			+			+		+2.4		
*Piskurozyma fildesensis*	+	+					+								
*Vishniacozyma carnescens*	+1.1	+			+			+1.4	+2.3					+2.0	
*Vishniacozyma tephrensis*							+	−1.8	+		−1.9		+		
*Vishniacozyma victoriae*	+2.2	+1.6						+	+1.3				+	−1.1	

Peptide peaks appeared in ≥9 yeast species were shown in the table (m/z deviation ≤ 10 Da). + represent within a m/z deviation range of ±1 Da.

**Table 3:** Peptide markers of *Glaciozyma*, *Leucosporidium*, and *Phenoliferia* species in Microbotryomycetes.

*Glaciozyma antarctica*	*Glaciozyma litoralis*	*Glaciozyma martinii*	*Glaciozyma watsonii*	*Leucosporidium muscorum*	*Leucosporidium scottii*	*Phenoliferia glacialis*	*Phenoliferia psychrophenolica*	*Phenoliferia psychrophila*
2092.47	2109.13	2293.27	3064.02	2091.19	7222.34	2093.77	2092.42	2092.31
2104.64	2291.73	3062.47	3380.39	2104.71	8734.66	2106.54	2105.78	2105.08
3061.79	3062.39	3222.26	3465.01	4269.44		2294.39	2293.31	3019.34^#^
3221.38	3673.96	3377.06	3675.93	7218.59		3019.30^#^	3019.59^#^	3075.25
3378.45	5981.46	3674.43	6084.50			3076.19	3075.89	3221.94
3463.93	6108.28	5979.76	6125.95			3222.34	3222.12	3463.21
3673.92	6082.18	6085.23	6160.59^*^			3376.43	3376.88	3709.94^#^
4271.97	6123.42	6123.84	6799.21^*^			3463.91	3463.84	4271.45
5980.21	6160.09^*^	6161.06^*^	7349.51			3671.30	3709.41^#^	6107.53
6082.06	6799.44^*^	6799.68^*^	7775.31			3708.93^#^	4272.03	6149.22
6123.79	7222.73	7347.16	8735.44			6107.04	6109.20	6441.97
6160.07^*^	7346.55					6148.97	6150.87	6924.61
6799.33^*^	8734.66					6441.05	6443.32	7222.46
7347.03						6924.09	6926.67	7415.55
7775.13						7415.01	7417.46	7777.35
8735.67							7774.92	

Peptide peaks appeared in ≥3 yeast species in Microbotryomycetes were shown in the table (m/z deviation ≤ 5 Da).

* *Glaciozyma* genus-specific marker *(m/z deviation ≤ 2 Da)*.

# *Phenoliferia* genus-specific marker (m/z deviation ≤ 2 Da).

The majority of peptide markers for the 33 yeast species were in the range of *m/z* 5000–8000 (Table 2), indicating that proteins in this range were more stable and reproducible. Several peptide markers were determined in three psychrophilic yeasts (e.g., *Naganishia*-specific *m/z* 2980.15, *Dioszegia*-specific *m/z* 1106.6, and *Rhodotorula*-specific *m/z* 1230.4; Dalluge et al., 2019). The *m/z* value is only a simple characteristic of an individual protein, and many sequentially different proteins may produce similar *m/z* values (Spinali et al., 2015). Therefore, the yeast-specific peptide markers detected by MALDI-TOF MS should be further analyzed by other methods (e.g., protein sequencing).

### Comparison of Ethanol-Formic Acid Extraction Method and On-Plate Extraction Method for MALDI-TOF MS-Based Identification

The identification rate (%) was defined as the number of correct species identifications represented in the supplementary database relative to the total number of tested yeast samples. The calculation revealed that 33 (score > 9.0) out of the 33 yeast species in the supplementary database had an identification rate of 100%, meaning that all yeast samples using the ethanol-formic acid extraction method were correctly identified (Table 1). However, the identification rate was 87.88% (29 yeast species) using the on-plate extraction method in the present study (Table 1). Four yeast species were not identified to the species level using the on-plate extraction method, including *Leucosporidium muscorum*, *Phenoliferia psychrophila*, and *Filobasidium magnum* to genus level (score 6.0–9.0), and *Naganishia albidosimilis* to unidentified level (score < 6.0). Based on the analysis of morphological characteristics, we found that these species were mucoid and glistening. Mucoid growth is usually related to the encapsulation of yeast cells from the production of extracellular polysaccharides (Kurtzman et al., 2011), which might protect yeast cells and result in insufficient extraction.

Several extraction methods were investigated to extract peptides from cell colonies prior to analysis by MALDI-TOF MS, including on-plate extraction method, ethanol-formic acid extraction method, and trifluoroacetic acid extraction method (Rahi et al., 2016). The highest quality mass spectra were acquired using the ethanol-formic acid extraction method in the present study. Ethanol is a lipid membrane solvent which can help cells to distribute into the growing crystal, release intercellular proteins, and avoid producing excess chemical noise for MALDI-TOF MS analysis (Madonna et al., 2000). The addition of formic acid can modify the pH of the matrix solvent and thereby increase the spectra quality in the range of 2–12 kDa (Williams et al., 2003; Usbeck et al., 2013). In combination with the function of the above reagents, relatively high quality of MALDI-TOF MS data can be obtained. However, Wang et al. (2021) reported that the on-plate extraction method was more suitable and necessary for clinical identification, owing to its key advantages of simplicity and accuracy. These different effects of extraction methods on yeast identification may be partially due to the different species tested.

## Conclusion

With the development of mass spectrometry technology, different biomolecules and biological systems can be studied directly (Jang and Kim, 2018). However, in comparison with the clinical microbes, the MALDI-TOF MS data for environmental microbes are limited, especially yeasts in the polar extreme environments. Hitherto, most investigations on yeasts in polar areas have remained limited to their biodiversity and the quantification of rare or new species (Buzzini et al., 2017). Therefore, it is necessary to establish a MALDI-TOF MS database for polar yeast identification.

In this study, MALDI-TOF MS is demonstrated to be a suitable and accurate technology for the rapid identification and differentiation of yeast species from the polar regions. We have established a supplementary database including 33 yeast species from the polar regions in the current study. Compared with the ethanol-formic acid extraction method, the on-plate extraction method is quicker and easier but with low accuracy. More than 70% of polar yeast species were observed the same peptide markers, such as *m/z* 2091.8 and 2104.8, and the majority of peptide markers for the 33 yeast species appeared in the range of *m/z* 5000–8000. MALDI-TOF MS has potential application in microbial ecology studies, but standardized protocols and reference databases integration among laboratories is the main bottleneck at present (Lima et al., 2019). Therefore, these data are a foundation for the construction of a large-scale database of yeast species from the polar regions and other environments in the future.

## Data Availability Statement

The datasets presented in this study can be found in online repositories. The names of the repository/repositories and accession number(s) can be found in the article/Supplementary Material.

## Author Contributions

TZ and LY designed the study. CH conducted labwork, data analysis, and wrote the manuscript. JF conducted parts of data analysis. JS preserved the yeast strains. TZ revised the manuscript. All authors contributed to the article and approved the submitted version.

## Funding

This research was supported by National Natural Science Foundation of China (NSFC; Grant no. 31670025); CAMS Innovation Fund for Medical Sciences (Grant no. 2021-I2M-1-055); Projects of the Chinese Arctic and Antarctic Administration, State Oceanic Administration; and National Microbial Resource Center (Grant no. NMRC-2021-3).

## Conflict of Interest

The authors declare that the research was conducted in the absence of any commercial or financial relationships that could be construed as a potential conflict of interest.

## Publisher’s Note

All claims expressed in this article are solely those of the authors and do not necessarily represent those of their affiliated organizations, or those of the publisher, the editors and the reviewers. Any product that may be evaluated in this article, or claim that may be made by its manufacturer, is not guaranteed or endorsed by the publisher.

## Footnotes

^1^ http://www.cpcc.ac.cn

^2^ https://mafft.cbrc.jp/alignment/server/

## Abbreviations

MALDI-TOF MS: Matrix-assisted laser desorption/ionization time-of-flight mass spectrometry
MSPs: Main spectra profiles
LSU: Large subunit
ITS: Internal transcribed spacer
PCR: Polymerase chain reaction
AIC: Akaike information criteria
YMA: Yeast malt agar
CPCC: China Pharmaceutical Culture Collection
ML: Maximum Likelihood

## References

[ref1] Agustini B. C. Silva L. P. Bloch C. Bonfim T. M. da Silva G. A. (2014). Evaluation of MALDI-TOF mass spectrometry for identification of environmental yeasts and development of supplementary database. Appl. Microbiol. Biotechnol. 98, 5645–5654. doi: 10.1007/s00253-014-5686-7, PMID: 24687751

[ref2] Barahona S. Yuivar Y. Socias G. Alcaíno J. Cifuentes V. Baeza M. (2016). Identification and characterization of yeasts isolated from sedimentary rocks of union glacier at the Antarctica. Extremophiles 20, 479–491. doi: 10.1007/s00792-016-0838-6, PMID: 27215207

[ref3] Buzzini P. Branda E. Goretti M. Turchetti B. (2012). Psychrophilic yeasts from worldwide glacial habitats: diversity, adaptation strategies and biotechnological potential. FEMS Microbiol. Ecol. 82, 217–241. doi: 10.1111/j.1574-6941.2012.01348.x, PMID: 22385361

[ref4] Buzzini P. Turk M. Perini L. Turchetti B. Gunde-Cimerman N. (2017). “Yeasts in polar and subpolar habitats,” in Yeasts in Natural Ecosystems: Diversity. eds. Buzzini P. Lachance M. A. Yurkov A. (Cham: Springer), 331–365.

[ref5] Coutinho J. O. P. A. Peixoto T. S. de Menezes G. C. A. Carvalho C. R. Ogaki M. B. Gomes E. C. Q.. (2021a). In vitro and in vivo evaluation of the probiotic potential of Antarctic yeasts. Probiotics Antimicrob. Proteins 13, 1338–1354. doi: 10.1007/s12602-021-09758-8, PMID: 33759043

[ref6] Coutinho J. O. P. A. Quintanilha M. F. Campos M. R. A. Ferreira E. de Menezes G. C. A. Rosa L. H.. (2021b). Antarctic strain of *Rhodotorula mucilaginosa* UFMGCB 18, 377 attenuates mucositis induced by 5-fluorouracil in mice. Probiotics Antimicrob. Proteins. doi: 10.1007/s12602-021-09817-0 [Epub ahead of print] 34255281

[ref8] Dalluge J. J. Brown E. C. Connell L. B. (2019). Toward a rapid method for the study of biodiversity in cold environments: the characterization of psychrophilic yeasts by MALDI-TOF mass spectrometry. Extremophiles 23, 461–466. doi: 10.1007/s00792-019-01097-5, PMID: 31089891

[ref9] Fernandez P. M. Martorell M. M. Blaser M. G. Ruberto L. A. M. de Figueroa L. I. C. Mac Cormack W. P. (2017). Phenol degradation and heavy metal tolerance of Antarctic yeasts. Extremophiles 21, 445–457. doi: 10.1007/s00792-017-0915-5, PMID: 28271165

[ref10] Florio W. Tavanti A. Barnini S. Ghelardi E. Lupetti A. (2018). Recent advances and ongoing challenges in the diagnosis of microbial infections by MALDI-TOF mass spectrometry. Front. Microbiol. 9:1097. doi: 10.3389/fmicb.2018.01097, PMID: 29896172 PMC5986882

[ref11] Gardes M. Bruns T. D. (1993). ITS primers with enhanced specificity for basidiomycetes—application to identification of mycorrhizae and rusts. Mol. Ecol. 2, 113–118. doi: 10.1111/j.1365-294X.1993.tb00005.x, PMID: 8180733

[ref12] Jang K. S. Kim Y. H. (2018). Rapid and robust MALDI-TOF MS techniques for microbial identification: a brief overview of their diverse applications. J. Microbiol. 56, 209–216. doi: 10.1007/s12275-018-7457-0, PMID: 29492868

[ref13] Jin L. Cao J. R. Xue X. Y. Wu H. Wang L. F. Guo L.. (2020). Clinical and microbiological characteristics of *Cryptococcus gattii* isolated from 7 hospitals in China. BMC Microbiol. 20:73. doi: 10.1186/s12866-020-01752-4, PMID: 32228457 PMC7106762

[ref14] Kalyaanamoorthy S. Minh B. Q. Wong T. K. F. von Haeseler A. Jermiin L. S. (2017). ModelFinder: fast model selection for accurate phylogenetic estimates. Nat. Methods 14, 587–589. doi: 10.1038/nmeth.4285, PMID: 28481363 PMC5453245

[ref15] Kurtzman C. P. Fell J. W. Boekhout T. Robert V. (2011). “Methods for isolation, phenotypic characterization and maintenance of yeasts,” in The Yeasts: A Taxonomic Study. 5th *Edn*. eds. Kurtzman C. P. Fell J. W. Boekhout T. (Amsterdam: Elsevier), 88–107.

[ref16] Kurtzman C. P. Robnett C. J. (1991). Phylogenetic relation among species of *Saccharomyces*, *Schizosaccharomyces*, *Debaryomyces* and *Schwanomyces* determined from partial ribosomal RNA sequences. Yeast 7, 167–172. doi: 10.1002/yea.320070107, PMID: 2021083

[ref17] Li A. H. Yuan F. X. Groenewald M. Bensch K. Yurkov A. M. Li K.. (2020). Diversity and phylogeny of basidiomycetous yeasts from plant leaves and soil: proposal of two new orders, three new families, eight new genera and one hundred and seven new species. Stud. Mycol. 96, 17–140. doi: 10.1016/j.simyco.2020.01.002, PMID: 32206137 PMC7082220

[ref18] Lima M. S. de Lucas R. C. Lima N. Polizeli M. L. T. M. Santos C. (2019). Fungal community ecology using MALDI-TOF MS demands curated mass spectral databases. Front. Microbiol. 10:315. doi: 10.3389/fmicb.2019.00315, PMID: 30873137 PMC6401475

[ref19] Liu X. Z. Wang Q. M. Theelen B. Groenewald M. Bai F. Y. Boekhout T. (2015). Phylogeny of tremellomycetous yeasts and related dimorphic and filamentous basidiomycetes reconstructed from multiple gene sequence analyses. Stud. Mycol. 81, 1–26. doi: 10.1016/j.simyco.2015.08.001, PMID: 26955196 PMC4777771

[ref20] Madonna A. J. Basile F. Ferrer I. Meetani M. A. Rees J. C. Voorhees K. J. (2000). On-probe sample pretreatment for the detection of proteins above 15 KDa from whole cell bacteria by matrix-assisted laser desorption/ionization time-of-flight mass spectrometry. Rapid Commun. Mass Spectrom. 14, 2220–2229. doi: 10.1002/1097-0231(20001215)14:23<2220::AID-RCM155>3.0.CO;2-4, PMID: 11114032

[ref21] Martínez-Ávila L. Peidro-Guzmán H. Pérez-Llano Y. Moreno-Perlín T. Sánchez-Reyes A. Aranda E.. (2021). Tracking gene expression, metabolic profiles, and biochemical analysis in the halotolerant basidiomycetous yeast *Rhodotorula mucilaginosa* EXF-1630 during benzo[a]pyrene and phenanthrene biodegradation under hypersaline conditions. Environ. Pollut. 271:116358. doi: 10.1016/j.envpol.2020.116358, PMID: 33385892

[ref22] Martins P. M. M. Batista N. N. Miguel M. Simão J. B. P. Soares J. R. Schwan R. F. (2020). Coffee growing altitude influences the microbiota, chemical compounds and the quality of fermented coffees. Food Res. Int. 129:108872. doi: 10.1016/j.foodres.2019.108872, PMID: 32036899

[ref23] Moothoo-Padayachie A. Kandappa H. R. Krishna S. B. N. Maier T. Govender P. (2013). Biotyping *Saccharomyces cerevisiae* strains using matrix-assisted laser desorption/ionization time-of-flight mass spectrometry (MALDI-TOF MS). Eur. Food Res. Technol. 236, 351–364. doi: 10.1007/s00217-012-1898-1

[ref24] Nizovoy P. Bellora N. Haridas S. Sun H. Daum C. Barry K.. (2021). Unique genomic traits for cold adaptation in *Naganishia vishniacii*, a polyextremophile yeast isolated from Antarctica. FEMS Yeast Res. 21:foaa056. doi: 10.1093/femsyr/foaa056, PMID: 33232451

[ref25] Patel R. (2019). A moldy application of MALDI: MALDI-ToF mass spectrometry for fungal identification. J. Fungi 5:4. doi: 10.3390/jof5010004, PMID: 30609833 PMC6463175

[ref26] Pathan A. A. K. Bhadra B. Begum Z. Shivaji S. (2010). Diversity of yeasts from puddles in the vicinity of midre lovénbreen glacier, arctic and bioprospecting for enzymes and fatty acids. Curr. Microbiol. 60, 307–314. doi: 10.1007/s00284-009-9543-3, PMID: 19967375

[ref27] Paul S. Singh P. Sharma S. Prasad G. S. Rudramurthy S. M. Chakrabarti A.. (2019). MALDI-TOF MS-based identification of melanized fungi is faster and reliable after the expansion of in-house database. Proteomics Clin. Appl. 13:e1800070. doi: 10.1002/prca.201800070, PMID: 30141266

[ref28] Pavlova K. Panchev I. Krachanova M. Gocheva M. (2009). Production of an exopolysaccharide by Antarctic yeast. Folia Microbiol. 54, 343–348. doi: 10.1007/s12223-009-0049-y, PMID: 19826922

[ref29] Pavlovic M. Mewes A. Maggipinto M. Schmidt W. Messelhäußer U. Balsliemke J.. (2014). MALDI-TOF MS based identification of food-borne yeast isolates. J. Microbiol. Methods 106, 123–128. doi: 10.1016/j.mimet.2014.08.021, PMID: 25193440

[ref30] Pote S. T. Sonawane M. S. Rahi P. Shah S. R. Shouche Y. S. Patole M. S.. (2020). Distribution of pathogenic yeasts in different clinical samples: their identification, antifungal susceptibility pattern, and cell invasion assays. Infect Drug Resist. 13, 1133–1145. doi: 10.2147/IDR.S238002, PMID: 32368104 PMC7182453

[ref31] Rahi P. Prakash O. Shouche Y. S. (2016). Matrix-assisted laser desorption/ionization time-of-flight mass-spectrometry (MALDI-TOF MS) based microbial identifications: challenges and scopes for microbial ecologists. Front. Microbiol. 7:1359. doi: 10.3389/fmicb.2016.01359, PMID: 27625644 PMC5003876

[ref32] Rambaut A. (2018). Figtree version 1.4.4. Available at: https://github.com/rambaut/figtree (Accessed November 26, 2018).

[ref33] Spinali S. van Belkum A. Goering R. V. Girard V. Welker M. Van Nuenen M.. (2015). Microbial typing by matrix-assisted laser desorption ionization-time of flight mass spectrometry: do we need guidance for data interpretation? J. Clin. Microbiol. 53, 760–765. doi: 10.1128/JCM.01635-14, PMID: 25056329 PMC4390642

[ref34] Spitaels F. Wieme A. D. Janssens M. Aerts M. Van Landschoot A. De Vuyst L.. (2015). The microbial diversity of an industrially produced lambic beer shares members of a traditionally produced one and reveals a core microbiota for lambic beer fermentation. Food Microbiol. 49, 23–32. doi: 10.1016/j.fm.2015.01.008, PMID: 25846912

[ref35] Stamatakis A. (2014). RAxML version 8: a tool for phylogenetic analysis and post-analysis of large phylogenies. Bioinformatics 30, 1312–1313. doi: 10.1093/bioinformatics/btu033, PMID: 24451623 PMC3998144

[ref36] Tan K. E. Ellis B. C. Lee R. Stamper P. D. Zhang S. X. Carroll K. C. (2012). Prospective evaluation of a matrix-assisted laser desorption ionization-time of flight mass spectrometry system in a hospital clinical microbiology laboratory for identification of bacteria and yeasts: a bench-by-bench study for assessing the impact on time to identification and cost-effectiveness. J. Clin. Microbiol. 50, 3301–3308. doi: 10.1128/JCM.01405-12, PMID: 22855510 PMC3457442

[ref37] Tsuji M. (2017). Genetic diversity of yeasts from East Ongul Island, East Antarctica and their extracellular enzymes secretion. Polar Biol. 41, 249–258. doi: 10.1007/s00300-017-2185-1

[ref38] Usbeck J. C. Kern C. C. Vogel R. F. Behr J. (2013). Optimization of experimental and modelling parameters for the differentiation of beverage spoiling yeasts by matrix-assisted-laser-desorption/ionization-time-of-flight mass spectrometry (MALDI-TOF MS) in response to varying growth conditions. Food Microbiol. 36, 379–387. doi: 10.1016/j.fm.2013.07.004, PMID: 24010620

[ref39] Usbeck J. C. Wilde C. Bertrand D. Behr J. Vogel R. F. (2014). Wine yeast typing by MALDI-TOF MS. Appl. Microbiol. Biotechnol. 98, 3737–3752. doi: 10.1007/s00253-014-5586-x, PMID: 24615383

[ref40] Viñarta S. C. Angelicola M. V. Van Nieuwenhove C. Aybar M. J. de Figueroa L. I. C. (2020). Fatty acids profiles and estimation of the biodiesel quality parameters from *Rhodotorula* spp. from Antarctica. Biotechnol. Lett. 42, 757–772. doi: 10.1007/s10529-020-02796-2, PMID: 31997042

[ref41] Walsh P. S. Metzger D. A. Higuchi R. (1991). Chelex 100 as a medium for simple extraction of DNA for PCR-based typing from forensic material. Biotechniques 10, 506–513. doi: 10.2144/000114018, PMID: 1867860

[ref42] Wang Q. M. Begerow D. Groenewald M. Liu X. Z. Theelen B. Bai F. Y.. (2015). Multigene phylogeny and taxonomic revision of yeasts and related fungi in the *Ustilaginomycotina*. Stud. Mycol. 81, 55–83. doi: 10.1016/j.simyco.2015.10.004, PMID: 26955198 PMC4777779

[ref43] Wang Y. Le L. T. H. L. Yoo W. Lee C. W. Kim K. K. Lee J. H.. (2019). Characterization, immobilization, and mutagenesis of a novel cold-active acetylesterase (EaAcE) from *Exiguobacterium antarcticum* B7. Int. J. Biol. Macromol. 136, 1042–1051. doi: 10.1016/j.ijbiomac.2019.06.108, PMID: 31229546

[ref44] Wang J. Wang H. Cai K. Yu P. Liu Y. Zhao G.. (2021). Evaluation of three sample preparation methods for the identification of clinical strains by using two MALDI-TOF MS systems. J. Mass Spectrom. 56:e4696. doi: 10.1002/jms.4696, PMID: 33421261 PMC7900945

[ref45] White T. J. Bruns T. Lee S. J. W. T. Taylor J. L. (1990). “Amplification and direct sequencing of fungal ribosomal RNA genes for phylogenetics,” in PCR Protocols: a guide to methods and applications. eds. Innis M. A. Gelfand D. H. Sninsky J. J. White T. J. (New York: Academic Press), 315–322.

[ref46] Wigmann É. F. Behr J. Vogel R. F. Niessen L. (2019). MALDI-TOF MS fingerprinting for identification and differentiation of species within the *Fusarium fujikuroi* species complex. Appl. Microbiol. Biotechnol. 103, 5323–5337. doi: 10.1007/s00253-019-09794-z, PMID: 31037383

[ref47] Williams T. L. Andrzejewski D. Lay J. O. Musser S. M. (2003). Experimental factors affecting the quality and reproducibility of MALDI TOF mass spectra obtained from whole bacteria cells. J. Am. Soc. Mass Spectrom. 14, 342–351. doi: 10.1016/S1044-0305(03)00065-5, PMID: 12686481

[ref48] Zhang J. Plowman J. E. Tian B. Clerens S. On S. L. W. (2020). An improved method for MALDI-TOF analysis of wine-associated yeasts. J. Microbiol. Methods 172:105904. doi: 10.1016/j.mimet.2020.105904, PMID: 32229264

[ref49] Zhang T. Zhang Y. Q. Liu H. Y. Su J. Zhao L. X. Yu L. Y. (2014). *Cryptococcus fildesensis* sp. nov., a psychrophilic basidiomycetous yeast isolated from Antarctic moss. Int. J. Syst. Evol. Microbiol. 64, 675–679. doi: 10.1099/ijs.0.054981-0, PMID: 24271212

