# Supplementary material for: Application of Matrix-Assisted Laser Desorption/Ionization Time-of-Flight Mass Spectrometry for the Rapid Identification of Yeast Species From Polar Regions
Source: Front Microbiol. 2022 Feb 23;13:832893. doi: 10.3389/fmicb.2022.832893 (PMC8905632; doi:10.3389/fmicb.2022.832893)
Supplement: Supplementary file 1 [file Data_Sheet_1.docx]

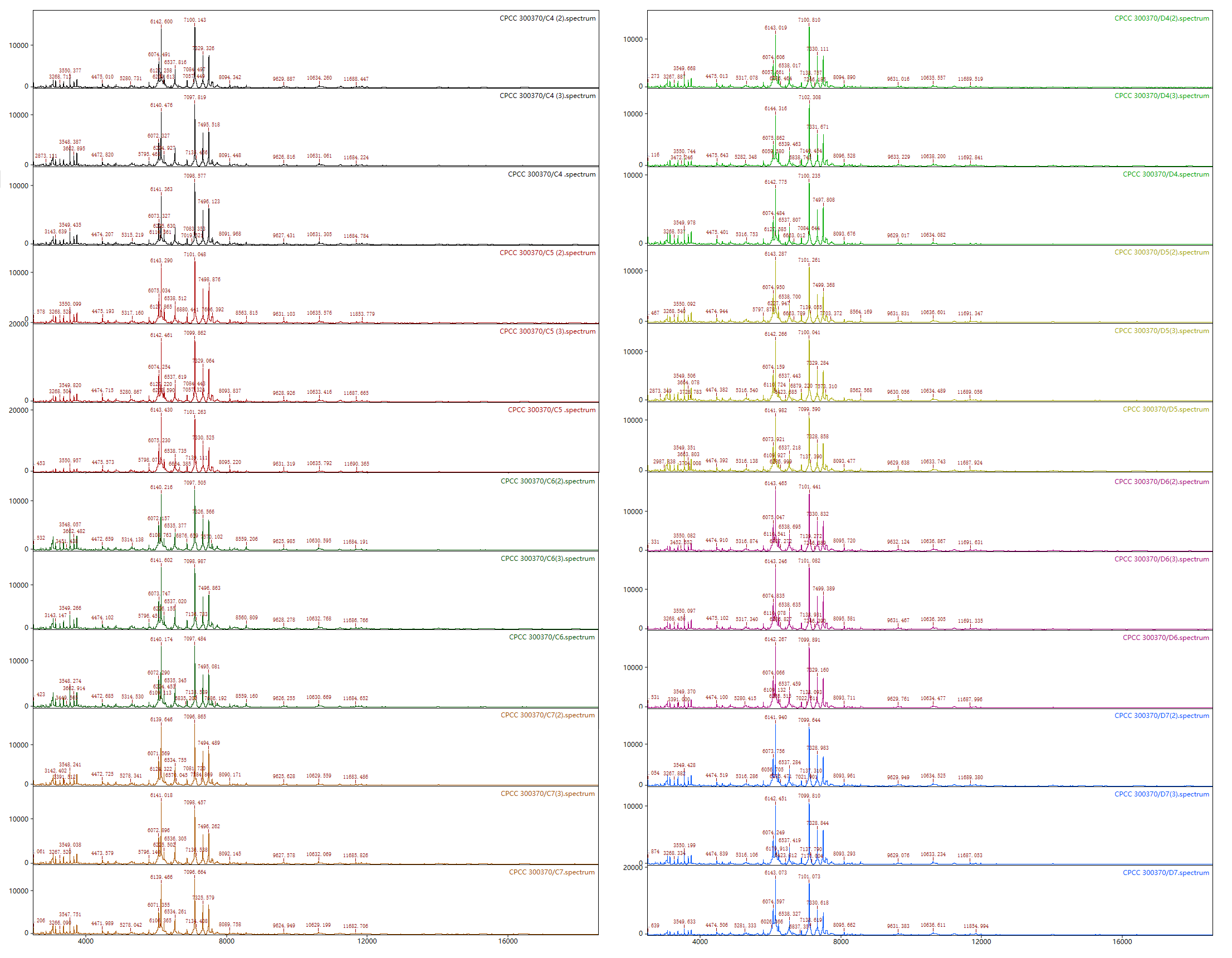


**Fig. S1** The 24 spectra profiles of *Cystobasidium laryngis* CPCC 300370 in the MALDI-TOF MS database.

**
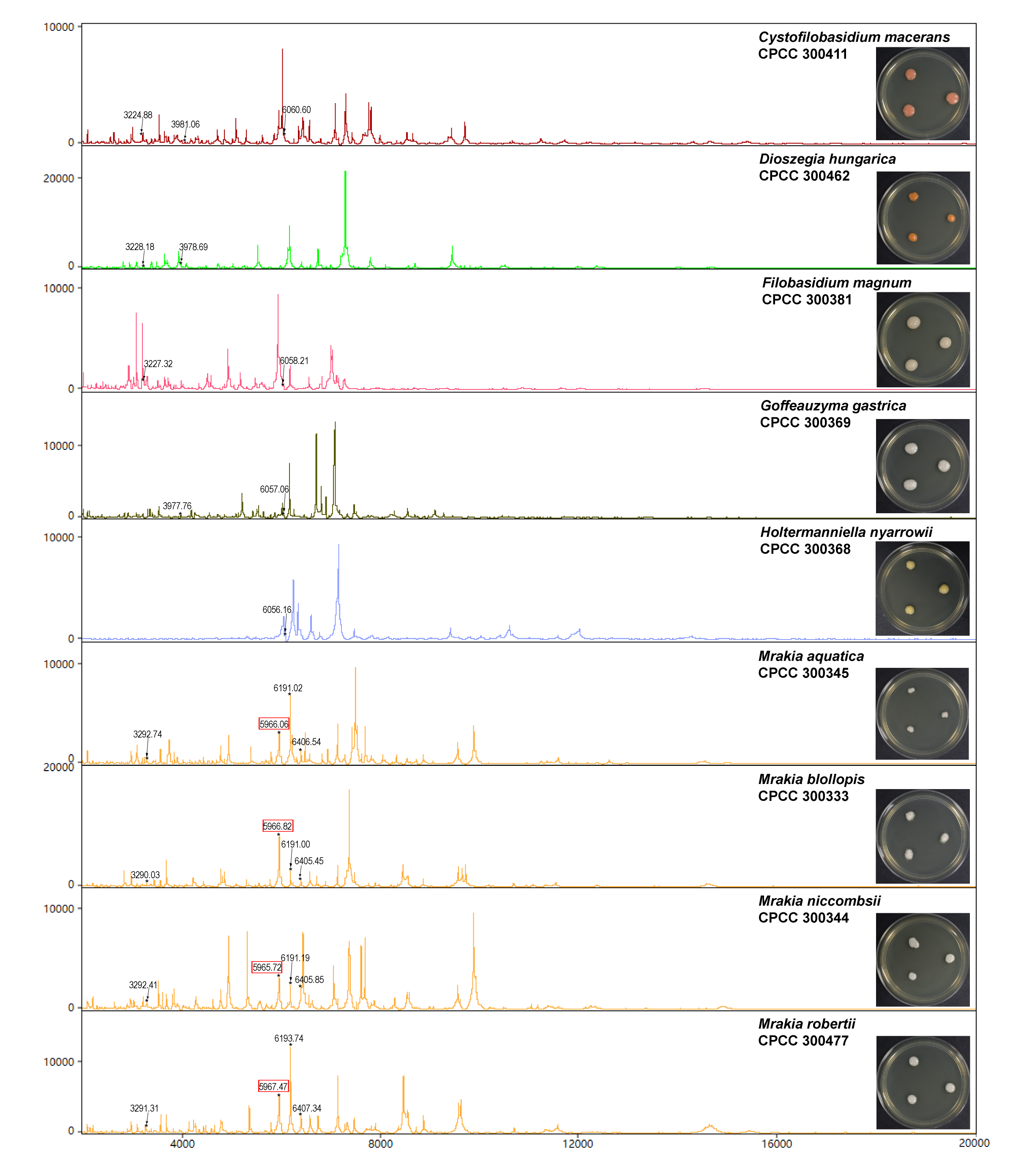
**

**
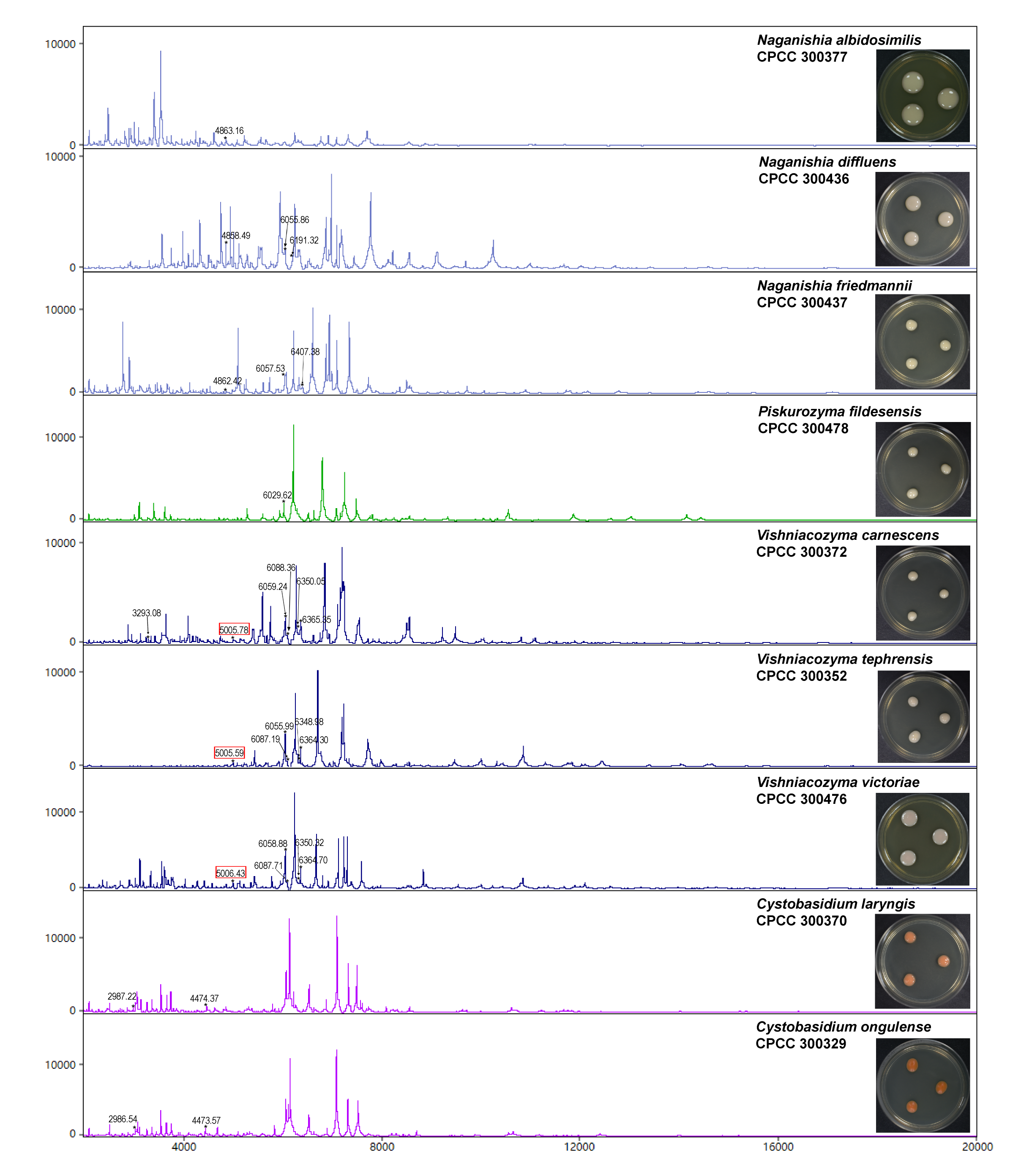
**

**Fig. S2** Representative mass spectra of the 18 yeast species in Tremellomycetes and Cystobasidiomycetes. The *Mrakia* genus-specific markers (about *m/z* 5966 Da) and *Vishniacozyma* genus-specific markers (about *m/z* 5006 Da) were marked by red quadrangles, respectively.


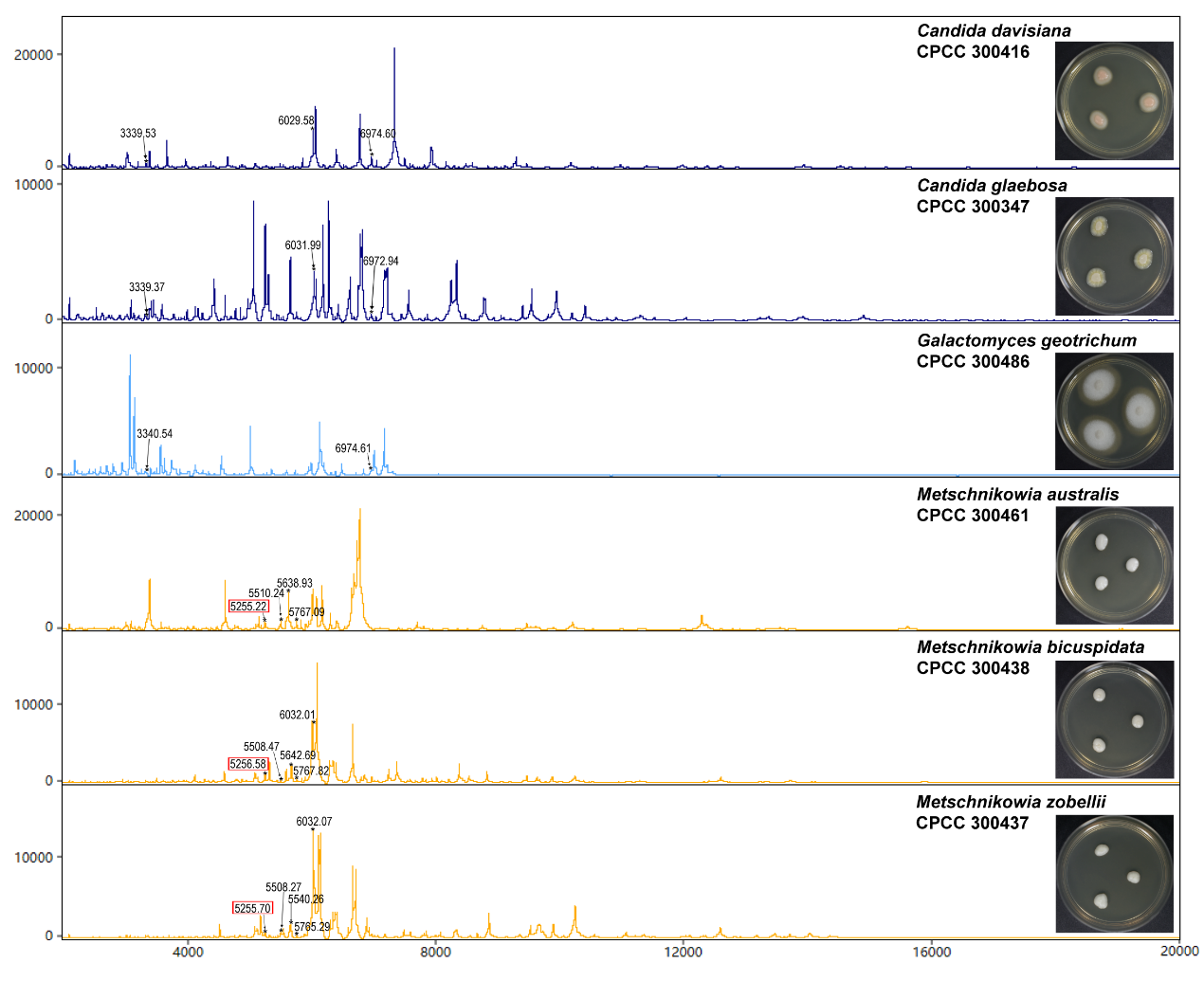


**Fig. S3** Representative mass spectra of the six yeast species in Saccharomycetes. The *Metschnikowia* genus-specific markers (about *m/z* 5256 Da) were marked by red quadrangles.

**Table S1** Peptide peaks of *Mrakia*, *Naganishia* and *Vishniacozyma* species in Tremellomycetes.

| *M. aquatica* | *M. blollopis* | *M. niccombsii* | *M. robertii* | *N. albidosimilis* | *N. diffluens* | *N. friedmannii* | *V. carnescens* | *V. tephrensis* | *V. victoriae* |
| --- | --- | --- | --- | --- | --- | --- | --- | --- | --- |
| 2089.41 | 2089.95 | 2089.63 | 2091.75 | 2090.17 | 4858.49 | 2092.59 | 2092.81 | 5005.59^#^ | 2094.06 |
| 2102.55 | 2102.65 | 2103.38 | 2104.81 | 2100.53 | 6055.86 | 2107.26 | 2105.78 | 6055.99 | 2106.27 |
| 3292.74 | 3290.03 | 2639.00 | 3291.22 | 2642.07 | 6084.29 | 2641.89 | 3293.08 | 6087.19 | 3030.92 |
| 5966.06* | 4278.09 | 3030.77 | 3976.91 | 3026.04 | 6191.32 | 3975.99 | 3976.42 | 6180.62 | 5006.43^#^ |
| 6191.02 | 4861.74 | 3292.41 | 4276.81 | 4277.99 | 6814.89 | 4862.42 | 5005.78^#^ | 6348.98 | 6058.88 |
| 6406.54 | 5966.82* | 5965.72* | 5967.47* | 4863.16 | 7722.60 | 6057.53 | 6059.24 | 6364.40 | 6087.71 |
| 7127.16 | 6191.00 | 6191.19 | 6193.74 | 7327.14 | 8542.24 | 6182.84 | 6088.36 | 6404.90 | 6350.32 |
| 7143.51 | 6406.45 | 6405.85 | 6364.03 | 8554.50 | 8558.86 | 6407.38 | 6179.63 | 6818.30 | 6364.70 |
| 7723.65 | 7101.93 | 6814.82 | 6407.34 |  |  |  | 6350.05 | 8555.81 | 7127.42 |
| 7817.56 | 7126.75 | 7101.84 | 7104.77 |  |  |  | 6365.35 |  | 7818.37 |
| 8543.46 | 7142.85 | 7143.29 | 7328.02 |  |  |  | 7299.89 |  |  |
|  | 7299.50 | 7299.53 | 8554.51 |  |  |  | 7722.48 |  |  |
|  | 7327.64 | 7722.89 |  |  |  |  | 8560.29 |  |  |
|  | 8557.90 | 7817.52 |  |  |  |  |  |  |  |
|  |  | 8543.37 |  |  |  |  |  |  |  |

*Peptide peaks appeared in ≥ three yeast species in Tremellomycetes were shown in the table (m/z deviation ≤ 5 Da). * Mrakia genus-specific marker; # Vishniacozyma genus-specific marker (m/z deviation ≤ 2 Da).*

**Table S2** Peptide peaks of *Candida*, *Galactomyces*, and *Metschnikowia* species in Saccharomycetes.

| *C. davisiana* | *C. glaebosa* | *G. geotrichum* | *M. australis* | *M. bicuspidata* | *M. zobellii* |
| --- | --- | --- | --- | --- | --- |
| 2091.01 | 2091.59 | 3074.80 | 2091.89 | 5110.29 | 2092.69 |
| 2104.53 | 2104.84 | 3340.54 | 2104.52 | 5256.58* | 2105.46 |
| 3071.51 | 3339.37 | 6171.57 | 5110.84 | 5508.47 | 3074.83 |
| 3339.53 | 5665.71 | 6311.88 | 5255.22* | 5642.69 | 5255.70* |
| 5112.88 | 6031.99 | 6719.80 | 5510.24 | 5767.82 | 5508.27 |
| 6029.58 | 6153.49 | 6974.61 | 5638.93 | 6032.03 | 5640.26 |
| 6198.13 | 6170.34 |  | 5666.66 | 6150.60 | 5666.36 |
| 6300.81 | 6972.94 |  | 5767.09 | 6198.12 | 5765.29 |
| 6407.50 |  |  | 6152.47 | 6300.82 | 6032.16 |
| 6743.09 |  |  | 6201.38 | 6669.46 | 6172.96 |
| 6974.60 |  |  | 6313.03 | 6718.92 | 6301.82 |
| 9477.88 |  |  | 6407.97 | 9480.92 | 6313.96 |
|  |  |  | 6743.24 |  | 6411.74 |
|  |  |  | 9477.27 |  | 6669.76 |
|  |  |  |  |  | 6717.75 |
|  |  |  |  |  | 6744.96 |

*Peptide peaks appeared in ≥ three yeast species in Saccharomycetes were shown in the table (m/z deviation ≤ 5 Da). * Metschnikowia genus-specific marker (m/z deviation ≤ 2 Da).*
